# Exposure to Multiple Pesticides and Risk of Non-Hodgkin Lymphoma in Men from Six Canadian Provinces

**DOI:** 10.3390/ijerph8062320

**Published:** 2011-06-21

**Authors:** Karin Hohenadel, Shelley A. Harris, John R. McLaughlin, John J. Spinelli, Punam Pahwa, James A. Dosman, Paul A. Demers, Aaron Blair

**Affiliations:** 1 Occupational Cancer Research Centre, 505 University Avenue, 14th floor, Toronto, Ontario M5G 1X3, Canada; E-Mails: shelley.harris@cancercare.on.ca (S.A.H.); paul.demers@cancercare.on.ca; (P.A.D.); blairkansas@aol.com (A.B.); 2 Cancer Care Ontario, 505 University Avenue, 14th floor, Toronto, Ontario M5G 1X3, Canada; 3 Dalla Lana School of Public Health, University of Toronto, 155 College Street, 6th floor, Toronto, Ontario M5T 3M7, Canada; E-Mail: jmclaugh@lunenfeld.ca (J.R.M.); 4 Samuel Lunenfeld Research Institute, Mount Sinai Hospital, 60 Murray Street, Toronto, Ontario M5G 1X5, Canada; 5 BC Cancer Agency, 675 West 10th Avenue, Vancouver, British Columbia V5Z 1L3, Canada; E-Mail: jspinelli@bccrc.ca; 6 School of Population and Public Health, University of British Columbia, 2206 East Mall, Vancouver, British Columbia V6T 1Z3, Canada; 7 Department of Community Health and Epidemiology, University of Saskatchewan, Health Science Building, 107 Wiggins Road, Saskatoon, Saskatchewan S7N 5E5, Canada; E-Mail: pup165@mail.usask.ca; 8 Canadian Centre for Health and Safety in Agriculture, Royal University Hospital, University of Saskatchewan, Room 3608, Wing 3E, 103 Hospital Drive, Saskatoon, Saskatchewan S7N 0W8, Canada; E-Mail: james.dosman@usask.ca

**Keywords:** occupational cancer, non-Hodgkin lymphoma, pesticides, case-control study

## Abstract

Non-Hodgkin lymphoma (NHL) has been linked to several agricultural exposures, including some commonly used pesticides. Although there is a significant body of literature examining the effects of exposure to individual pesticides on NHL, the impact of exposure to multiple pesticides or specific pesticide combinations has not been explored in depth. Data from a six-province Canadian case-control study conducted between 1991 and 1994 were analyzed to investigate the relationship between NHL, the total number of pesticides used and some common pesticide combinations. Cases (n = 513) were identified through hospital records and provincial cancer registries and controls (n = 1,506), frequency matched to cases by age and province of residence, were obtained through provincial health records, telephone listings, or voter lists. In multiple logistic regression analyses, risk of NHL increased with the number of pesticides used. Similar results were obtained in analyses restricted to herbicides, insecticides and several pesticide classes. Odds ratios increased further when only ‘potentially carcinogenic’ pesticides were considered (OR[one pesticide] = 1.30, 95% CI = 0.90–1.88; OR[two to four] = 1.54, CI = 1.11–2.12; OR[five or more] = 1.94, CI = 1.17–3.23). Elevated risks were also found among those reporting use of malathion in combination with several other pesticides. These analyses support and extend previous findings that the risk of NHL increases with the number of pesticides used and some pesticide combinations.

## 1. Introduction

Non-Hodgkin lymphoma (NHL) has been associated with several agricultural and farm-specific exposures, including some phenoxy herbicide, organochlorine, organophosphate and carbamate pesticides [1–3]. Although a number of studies have examined the relationship between individual pesticides and NHL, few studies investigate the impact of exposure to multiple pesticides or specific pesticide combinations. This is necessary because most pesticide applicators use multiple chemicals throughout the year or in combination for individual applications.

DeRoos and colleagues pooled data from three NHL case-control studies conducted in the 1980s in four American mid-western states in one of the first attempts to examine the impact of exposure to multiple pesticides [4]. They found that, although the risk of NHL increased marginally with the number of pesticides used, it increased substantially when analyses were restricted to ‘potentially carcinogenic’ pesticides. Further, they found a super-additive effect whereby use of atrazine amplified risk of NHL when used in combination with several other pesticides including alachlor, diazinon and carbofuran [4].

In order to further evaluate the findings reported by DeRoos [4] we used data from a multi-provincial Canadian study to examine the impact of exposure to multiple pesticides, and common use combinations of pesticides, on the risk of NHL [5].

## 2. Methods

### 2.1. Data Source

The data used in these analyses were part of the Cross-Canada Study of Pesticides and Health, a case-control study of Canadian men 19 years of age or older, conducted between 1991 and 1994 in six Canadian provinces (Alberta, British Columbia, Manitoba, Ontario, Quebec, and Saskatchewan) [5]. Cases of NHL, Hodgkin lymphoma, soft tissue sarcoma, and multiple myeloma were identified through hospital records in Quebec and from cancer registries in all other provinces. A common control group for all cancer sites was assembled using provincial health insurance records (Alberta, Saskatchewan, Manitoba, and Quebec), computerized telephone listings (Ontario) and voter lists (British Columbia). Controls were frequency matched to cases by age (±2 years) and province of residence [5].

Information on demographic characteristics, medical and occupational history, exposure to selected substances, and other potentially confounding variables was obtained from all participants via a postal questionnaire. Detailed information on pesticide use was collected by telephone interview from all participants indicating they had ten or more hours of pesticide use during their lifetime and a 15% random sample of those with less than 10 hours. Specific pesticides were included in the questionnaire if the compound was ever registered for use in Canada and reviewed by the International Agency for Research on Cancer (IARC); if it was recently restricted or banned in Canada; or, if it was commonly used in Canada. Included pesticides were listed in table format, along with variables for number of days used and number of hours per day at home or work. This method of collecting pesticide use data was validated in a pilot study whereby twenty-seven volunteer farmers completed the questionnaire and subsequently provided purchase records. Investigators found excellent concordance between the two sources [5].

Questionnaires used in both portions of the study were modified versions of the questionnaire developed for a study of pesticide exposure, NHL and other tumors in Kansas and Nebraska, which were included in the analyses presented by DeRoos [4]. A detailed description of the data collection procedures for the Cross-Canada Study of Pesticides and Health has been published elsewhere [5,6]. The data used here are slightly different from previous publications because a pathology review resulted in the exclusion of four cases of NHL.

### 2.2. Statistical Analyses

#### 2.2.1. Exposure to Multiple Pesticides

A brief examination of the impact of exposure to multiple pesticides on NHL has been reported previously in this population [5]. To expand upon these analyses, the total number of pesticides individuals reported using was categorized into four groups: no pesticide use, and use of one, two to four, or five or more pesticides. Additional analyses were conducted looking at number of insecticides, herbicides and fungicides used; the number of phenoxy herbicides, organochlorines, and organophosphates used; and the number of ‘potentially carcinogenic’ pesticides used. A pesticide was considered ‘potentially carcinogenic’ if it was classified as possibly carcinogenic to humans (group 2B) or higher by IARC [7], or suggestive evidence of carcinogenic potential or more severe by the United States Environmental Protection Agency (US EPA) Integrated Risk Assessment System or Office of Pesticides Program [8,9] (for a complete list of pesticides determined to be ‘potentially carcinogenic’ see Appendix A). All analyses were conducted using the statistical package SAS, version 9.2. Trends were examined using the Cochrane-Armitage test. Dose and duration information were not utilized in this analysis due to sample size limitations, which restricted further stratification.

#### 2.2.2. Combinations of Pesticides

For the purpose of this analysis, a pesticide combination was defined as any two pesticides used by the same person. Commonly used pesticide combinations were determined by generating a correlation matrix of all pesticides used by twenty or more participants. All combinations yielding a correlation coefficient of 0.4 or greater were examined. In addition, combinations containing either malathion or mecoprop with a correlation coefficient of 0.3 or greater were examined based on hypotheses generated from associations found in preliminary analyses conducted using this dataset.

Unconditional logistic regression models were generated with variables for use of either individual pesticide in the combination, use of both pesticides, and use of neither pesticide. Where the odds ratio for joint exposure was higher than the odds ratio for exposure to either pesticide in the combination alone, interaction on the additive scale was evaluated using an interaction contrast ratio (ICR = OR_both pesticides_ − OR_pesticide 1 only_ − OR_pesticide 2 only_ + 1). ICR values above 0.5 were interpreted as indicating super-additivity. Models were developed which include a variety of potentially confounding factors suggested by the literature, including exposure to diesel exhaust, ultra-violet rays, and chemicals such as benzene; and family history of cancer in a first-degree relative.

The University of Toronto Health Sciences Research Ethics Board reviewed and approved the protocol for these secondary analyses. Ethics approval for data collection in the original study was obtained from research ethics boards in each province.

## 3. Results

The dataset used in this analysis contains information on 513 NHL cases and 1,506 controls. This represents 66.6% of contacted cases and 48.0% of contacted controls. As reported by McDuffie *et al*., potential subjects from urban and rural areas were equally likely to respond, and a greater proportion of responders were in the middle-age group than at either extreme among both cases and controls [5].

Cases were slightly older than controls and, proportional to their population size, the greatest number of cases and controls were obtained from Ontario and Quebec (Table 1). Proxy respondents were required for 21% of the cases and 15% of the controls. Nearly half of the participants had lived or worked on a farm in their lifetime. Additional demographic information on the participants has been published previously [5].

### 3.1. Multiple Pesticides

Risk of NHL tended to be greater among individuals who reported use of an increasing number of any type of pesticide (Table 2). This pattern was also evident for subgroups of herbicides, insecticides and fungicides. Odds ratios in the highest pesticide use category were 1.63 (95% CI: 1.20–2.21, p[trend] = 0.01) for any pesticide, 1.57 (95% CI: 0.96–2.57, p[trend] = 0.02) for herbicides, 1.70 (95% CI: 0.95–3.05, p[trend] < 0.01) for insecticides and 1.72 (95% CI: 1.07–2.77, p[trend] = 0.04) for fungicides. Odds ratios were also typically elevated for the use category of two to four pesticides, but less so than in the upper category. NHL risk also increased with number of pesticides used by chemical class (Table 3). Odds ratios tended to be the largest among participants using two or more pesticides in these categories with 1.78 (95% CI: 1.27–2.50, p[trend] = 0.01) for phenoxy herbicides, 1.36 (95% CI: 0.92–2.02, p[trend] = 0.15) for organochlorines, and 1.69 (95% CI: 1.04–2.74, p[trend] < 0.01) for organophosphates.

When analyses were restricted to those pesticides determined to be ‘potentially carcinogenic’, odds ratios increased further to 1.30 (95% CI: 0.90–1.88) in those reporting use of one pesticide, 1.54 (95% CI: 1.11–2.12) in those using two to four pesticides and 1.94 (95% CI: 1.17–3.23) in those using five or more pesticides (p[trend] = 0.01) (Table 2). This odds ratio is greater than any produced when examining use of any single pesticide [5]. Odds ratios were not significantly impacted by adjusting for potentially confounding factors such as exposure to ultra-violet rays, farm animals, or diesel exhaust (not presented).

### 3.2. Combinations of Pesticides

The correlation matrix yielded thirty-six pesticide combinations for analysis (for complete list of combinations examined see Appendix B). Several pesticide combinations produced higher odds ratios among participants using both pesticides than those reporting use of either one (Tables 4). These combinations always included malathion: malathion and 2,4-D, malathion and mecoprop, malathion and glyphosate, malathion and DDT, and malathion and carbaryl. None of the interaction terms in these models were statistically significant, and only malathion and carbaryl had a super-additive joint effect (ICR > 0.5). Similar to analyses on multiple pesticides, these findings were not impacted by adjusting for potentially confounding factors.

## 4. Discussion

Investigations of pesticides and cancer have, quite appropriately, focused on potential effects of individuals chemicals whenever possible for ease of analysis and policy and regulation purposes. Multiple exposures, however, complicate assessment of relationships between pesticides and cancer and more accurately reflect how pesticides are used in practice. McDuffie [5] previously reported that the risk of NHL in the Cross-Canada Study of Pesticides and Health tended to increase with the number of pesticides used. In a study from the United States, DeRoos [4] reported similar results in some cases, noting that risk increases when only pesticides with some evidence of carcinogenicity were included in the analysis and that risk were also increased for several specific combinations. Our results extend these findings.

The risk of NHL rose with increasing numbers of pesticides used and tests for trend were almost always statistically significant. Two additional findings stand out. First, the rising trend did not appear to be associated with any particular pesticide class and was observed for herbicides, insecticides, and fungicides. These analyses, however, are not on mutually exclusive exposure groups because many individuals used pesticides from all three classes. Second, odds ratios increased further when only pesticides with some evidence of carcinogenicity were considered in the summation. Risk rose to nearly two-fold among those reporting use of five or more potentially carcinogenic pesticides.

Our findings and those from earlier studies [4,5] might be explained in a several ways. It could be that several pesticides each contribute a small risk that sums to a larger relative risk when they are considered in combination. Another explanation might be that as the number of pesticides used increases, the chances of including one or more that has considerable carcinogenic properties may also increase. Finally, use of multiple pesticides may be acting as a proxy measure for a more complex farming operation that may present some unique exposures that could be related to NHL.

DeRoos [4] had found that specific combinations of pesticides led to higher risks than would have been predicted from additive models, particularly those combinations that included atrazine. We were unable to evaluate findings for atrazine because its use was only reported by five individuals in the Cross-Canada Study of Pesticides and Health. Our analyses of specific combinations of pesticides did find some evidence of increased risk related to use of malathion in combination with 2,4-D, mecoprop, carbaryl, glyphosate, and DDT, where odds ratios increased beyond that from use of either pesticide alone. Interaction odds ratios should be interpreted cautiously because odds ratios for most combinations are not much larger than for malathion alone and were not statistically significant, and only the combination of malathion and carbaryl appeared to have a super-additive effect.

Findings indicating increased risk with reported use of pesticide combinations including malathion, a common organophosphate insecticide used on a wide range of crops and gardens and for public health-related mosquito control, are somewhat unexpected given that there is limited evidence of its carcinogenicity in human and animal studies. IARC categorized malathion as a group 3 substance (not classifiable as to its carcinogenicity to humans), and the US EPA classified it as having “suggestive evidence of carcinogenicity” [10,11]. There are several hypothesized mechanisms of carcinogenicity for malathion but they are not well-established, particularly for NHL [12].

A major limitation of our analysis is that our proxy measures for pesticide exposure were based on self-reported lifetime use. It is not clear whether use of combinations of pesticides were from actual tank mixtures, combinations used during the same growing season, or use in different years over a lifetime. These are quite different exposure scenarios and, even if the pesticides were carcinogenic, we might expect quite different biologic effects from these different exposure patterns. Moreover, we have no direct information on pesticide exposure or absorbed dose because analyses were based on self-reported pesticide use, which was measured in a binary fashion. This may result in exposure measurement error and depending on the underlying distribution of true exposure, and the presence of confounding and other factors, risk estimates can be biased in unpredictable ways.

Furthermore, recall bias for exposures is a concern in case-control studies because cases may have spent more time thinking about past exposures than controls. This could lead to differential misclassification and bias relative risks away from null. We lack direct information to address this issue, however, results from a methodological analysis of this issue in a similar case-control study in the United States did not uncover any evidence of case-response bias [13].

This study has several strengths. Information was obtained on pesticide use for a relatively large number of cases and controls. About 45% of cases and controls had lived or worked on a farm and occupational pesticide use was largely confined to this group. Accuracy of past events from questionnaires is always a concern, but farmer’s recall of pesticide has been found to be as good as for many other factors traditionally obtained by interview for epidemiologic studies [14]. Finally, information on many potential confounders for NHL was obtained and used in the models where appropriate but did not have a significant impact on risk.

## 5. Conclusions

These analyses confirm and extend previously reported results suggesting that the risk of NHL increases with the number of pesticides used, particularly when pesticides with some evidence of carcinogenicity are considered. Risk with reported use of combinations of pesticides showed few situations where risks were increased with pair wise use, although joint use of malathion and carbaryl appeared to have a super-additive effect. Additional work is needed to determine the role of exposure and dose, duration of exposure and factors modifying exposures such as protective clothing, respirators and glove use on these multiple-use situations.

## Figures and Tables

**Table 1.** Comparison of non-Hodgkin lymphoma cases and controls in the Cross-Canada Study of Pesticides and Health.

	**Cases (n = 513)**		**Controls (n = 1,506)**	
	**Mean**	**SD**	**Mean**	**SD**

Age	57.71	14.26	54.08	16.35

	**N**	**%**	**N**	**%**

Province				

Alberta	65	12.67	196	13.01
British Columbia	126	24.56	230	15.27
Manitoba	34	6.63	113	7.50
Ontario	142	27.68	585	38.84
Quebec	117	22.81	291	19.32
Saskatchewan	29	5.65	91	6.04

	**N**	**%**	**N**	**%**

Ever lived or worked on a farm				
Yes	235	45.81	673	44.69
No	278	54.19	833	55.31

Respondent				
Self-respondent	403	78.56	1286	85.39
Proxy respondent	110	21.44	220	14.61

**Table 2.** Effect of exposure to multiple pesticides by pesticide type and carcinogenicity on NHL.

	**Cases** **N (%)**	**Controls** **N (%)**	OR *	**95% CI**

All pesticides			p(trend) = **0.01**	
0	352 (68.62)	1,095 (72.71)	1.00	–
1	14 (2.73)	56 (3.72)	0.80	0.44–1.47
2–4	67 (13.06)	176 (11.69)	**1.39**	**1.02–1.91**
5+	80 (15.59)	179 (11.89)	**1.63**	**1.20–2.21**

Herbicides			p(trend) = **0.02**	
0	369 (71.93)	1,147 (76.16)	1.00	–
1	45 (8.77)	127 (8.43)	1.24	0.86–1.80
2–4	73 (14.23)	167 (11.09)	**1.62**	**1.18–2.22**
5+	26 (5.07)	65 (4.32)	1.57	0.96–2.57

Insecticides			p(trend) < **0.01**	
0	367 (71.54)	1,153 (76.56)	1.00	–
1	43 (8.38)	126 (8.37)	1.22	0.84–1.77
2–4	85 (16.57)	189 (12.55)	**1.67**	**1.25–2.24**
5+	18 (3.51)	38 (2.52)	1.70	0.95–3.05

Fungicides			p(trend) = **0.04**	
0	453 (88.30)	1,361 (90.37)	1.00	–
1	30 (5.85)	90 (5.98)	1.03	0.67–1.60
2+	30 (5.85)	55 (3.65)	**1.72**	**1.07–2.77**

	**Cases** **N (%)**	**Controls** **N (%)**	OR *	**95% CI**

‘Potentially carcinogenic’ pesticides			p(trend) = **0.01**	
0	374 (72.90)	1,164 (77.29)	1.00	**–**
1	46 (8.97)	132 (8.76)	1.30	0.90–1.88
2–4	67 (13.06)	160 (10.62)	**1.54**	**1.11–2.12**
5+	26 (5.07)	50 (3.32)	**1.94**	**1.17–3.23**

* Adjusted for age, province and use of a proxy respondent.

**Table 3.** Effect of exposure to multiple pesticides by selected classes on NHL.

	**Cases** **N (%)**	**Controls** **N (%)**	OR *	**95% CI**

Phenoxy herbicides			p(trend) = **0.01**	
0	384 (74.85)	1,188 (78.88)	1.00	–
1	66 (12.87)	185 (12.28)	1.33	0.97–1.82
2+	63 (12.28)	133 (8.83)	**1.78**	**1.27–2.50**

Organochlorines			p(trend) = 0.15	
0	407 (79.34)	1,230 (81.67)	1.00	–
1	66 (12.87)	169 (11.22)	1.33	0.97–1.81
2+	40 (7.80)	107 (7.10)	1.36	0.92–2.02

Organophosphates			p(trend) < **0.01**	
0	421 (82.07)	1,337 (88.78)	1.00	–
1	65 (12.67)	115 (7.64)	**2.10**	**1.50–2.94**
2+	27 (5.26)	54 (3.59)	**1.69**	**1.04–2.74**

* Adjusted for age, province and use of a proxy respondent.

**Table 4.** Individual and joint effects of commonly used pesticide combinations on NHL.

	**Cases** **N (%)**	**Controls** **N (%)**	OR *	**95% CI**

Malathion and 2,4-D			p = 0.59, ICR = 0.39	
Malathion only	11 (2.14)	21 (1.39)	1.73	0.81–3.66
2,4-D only	49 (9.55)	187 (12.42)	0.94	0.67–1.33
Malathion and 2,4-D	61 (11.89)	106 (7.04)	**2.06**	**1.45–2.93**

Malathion and carbaryl			p = 0.45, ICR = **1.42**	
Malathion only	52 (10.14)	106 (7.04)	**1.75**	**1.22–2.52**
Carbaryl only	5 (0.97)	13 (0.86)	1.17	0.41–3.36
Malathion and carbaryl	20 (3.90)	21 (1.39)	**3.34**	**1.77–6.31**

Malathion and DDT			p = 0.30, ICR = −0.64	
Malathion only	52 (10.14)	95 (6.31)	**2.03**	**1.41–2.94**
DDT only	13 (2.53)	27 (1.79)	1.72	0.86–3.42
Malathion and DDT	20 (3.90)	32 (2.12)	**2.11**	**1.17–3.80**

Malathion and glyphosate			p = 0.69, ICR = 0.23	
Malathion only	41 (7.99)	72 (4.78)	**1.95**	**1.29–2.93**
Glyphosate only	19 (3.70)	78 (5.18)	0.92	0.54–1.55
Malathion and glyphosate	31 (6.04)	55 (3.65)	**2.10**	**1.31–3.37**

Malathion and mecoprop			p = 0.64, ICR = 0.19	
Malathion only	44 (8.58)	92 (6.11)	**1.76**	**1.20–2.60**
Mecoprop only	23 (4.48)	46 (3.05)	**2.09**	**1.23–3.54**
Malathion and mecoprop	28 (5.46)	35 (2.32)	**3.04**	**1.80–5.15**

* Adjusted for age, province and use of a proxy respondent.

## Appendices

### Appendix A.

List of ‘potentially carcinogenic’ pesticides reportedly used by participants of the Cross-Canada Study of Pesticides and Health.

1. 2,4,5-T
2. 2,4-D
3. 2,4-DB
4. Arsenic
5. Asulam
6. Benomyl
7. Bromoxynil
8. Carbaryl
9. Cypermethrin
10. DDT
11. Dicamba
12. Diclofop-methyl
13. Dieldrin
14. Dimethoate
15. Dinoseb
16. Formaldehyde
17. Heptachlor
18. Lindane
19. Linuron
20. Mancozeb
21. MCPA
22. Mecoprop
23. Methidathion
24. Paraquat
25. Propoxur
26. Toxaphene
27. Triallate
28. Trichloroacetic acid
29. Trifluralin

### Appendix B.

Complete list of pesticide combinations evaluated.

1. Bromoxynil and diallate
2. Bromoxynil and glyphosate
3. Carbathin and bromoxynil
4. Carbathin and glyphosate
5. Carbofuran and diallate
6. Diallate and bromoxynil
7. Diallate and carbathin
8. Diclofop methyl and bromoxynil
9. Diclofop methyl and carbathin
10. Diclofop methyl and diallate
11. Difenzoquat and bromoxynil
12. Difenzoquat and carbathin
13. Difenzoquat and diclofop methyl
14. Difenzoquat and sethoxydim
15. Difenzoquat Trifluralin
16. Glyphosate and 2,4-D
17. Malathion and 2,4-D
18. Malathion and carbaryl
19. Malathion and DDT
20. Malathion and dimethoate
21. Malathion and glyphosate
22. Malathion and mecoprop
23. Malathion and methoxychlor
24. Mecoprop glyphosate
25. Mecoprop and methoxychlor
26. Mecoprop and 2,4-D
27. Methoxychlor and 2,4-D
28. Sethoxydim and bromoxynil
29. Sethoxydim and carbathin
30. Sethoxydim and carbofuran
31. Sethoxydim and diclofop-methyl
32. Triallate and diclofop-methyl
33. Triallate and trifluralin
34. Trifluralin and bromoxynil
35. Trifluralin and carbathin
36. Trifluralin and difenzoquat
